# Global perspectives on telemedicine-enabled medications for opioid use disorder: Practices, priorities, and barriers

**DOI:** 10.1177/1357633X251394442

**Published:** 2026-01-21

**Authors:** Joe Schofield, Alexander Mario Baldacchino, Atul Ambekar, Honest Anaba, Jenna L Butner, Nathaniel Day, Hamed Ekhtiari, Fatima Elomari, Marica Ferri, Konstantinos Kokkolis, Christos Kouimtsidis, Jonna Levola, Jiang Long, David Martell, Dario Gigena Parker, Afarin Rahimi-Movaghar, Kristiana Siste, Scott Steiger, Arash Khojasteh Zonoozi, Joseph Tay Wee Teck

**Affiliations:** 1 7486 School of Medicine, University of St Andrews, St Andrews, UK; 2 28730 National Drug Dependence Treatment Centre, All India Institute of Medical Sciences, New Delhi, India; 3 1466 Johns Hopkins University, Baltimore, USA; 4 5755 School of Medicine, Yale University, New Haven, USA; 5 Canadian Centre of Recovery Excellence, 3158 University of Alberta, Alberta, Canada; 6 5635 Department of Psychiatry & Behavioral Sciences, University of Minnesota, Minneapolis, USA; 7 Faculty of Medicine and Pharmacy, Mohammed Vth University of Rabat, Rabat, Morocco; 8 European Union Drugs Agency, Lisbon, Portugal; 9 743742 National Organisation for Prevention and Treatment of Addictions, Athens, Greece; 10 9490 Surrey and Borders Partnership NHS Trust, Leatherhead, UK; 11 3835 Department of Psychiatry, University of Helsinki and Helsinki University Hospital, Helsinki, Finland; 12 12474 Shanghai Mental Health Center, Shanghai Jiao Tong University School of Medicine, Shanghai, China; 13 3688 Dalhousie University, Nova Scotia, Canada; 14 National University of Córdoba, Córdoba, Argentina; 15 48439 Iranian National Center for Addiction Studies, Tehran University of Medical Sciences, Tehran, Iran; 16 64733 Department of Psychiatry, Faculty of Medicine, Universitas Indonesia – Dr. Cipto Mangunkusumo General Hospital, Jakarta, Indonesia; 17 8785 Department of Medicine, University of California, San Francisco, USA

**Keywords:** Telemedicine, addiction, opioid use disorder, global health, health equity

## Abstract

**Introduction:**

Telemedicine (TM) has potential to address the global opioid use disorder treatment gap, yet its uptake, priorities, and barriers have not been mapped internationally.

**Methods:**

We conducted a cross-sectional, web-based survey (July to November 2024) of clinicians and clinical leaders via the International Society of Addiction Medicine, World Psychiatric Association, and allied contacts. The questionnaire captured telemedicine facilitated medication for opioid use disorder (TMOUD) practices, priorities, and barriers. Responses were summarised overall and stratified by World Bank country-income group and by current TMOUD availability.

**Results:**

Sixty-eight experts from 37 countries, 32% from low/middle-income countries (LMICs), participated. General TM use rose from 57% before COVID-19 to 94% in 2024. TMOUD was available in 26 jurisdictions (38%), more often in high-income than LMIC settings (58% vs 11%). Barriers to prescribing were identified, and few settings reimbursed video and telephone consultations equally. Improving treatment retention (69%), reducing missed appointments (62%), and expanding medications to underserved (60%) or remote (57%) populations as top priorities, yet fewer than 40% reported that TMOUD was currently used to meet those goals. Key barriers were inadequate policy support (60%), lack of professional guidance (63%), restrictive regulation (48%), poor digital infrastructure (broadband 29%; e-prescribing 56%), and limited clinician training (54%); almost every barrier was more common in LMICs.

**Discussion:**

TMOUD remains uneven and concentrated in high-income countries. Updated clinical guidance, digital connectivity investment and interoperable e-health systems, and targeted workforce development, particularly in LMICs, are needed to realise TM's potential for equitable and effective treatment of opioid use disorder. This global survey fills a critical knowledge gap by documenting expert perspectives across income settings, offering cross-national evidence to inform equitable expansion of TMOUD worldwide.

## Introduction

### Opioid use disorder

Opioids produce analgesia and euphoria, and prolonged use can lead to tolerance and dependence. Opioid use disorder (OUD) is a chronic, relapsing condition characterised by an overpowering desire to use opioids, tolerance, continued use despite harm, and withdrawal during cessation. Globally, approximately 40 million people have OUD, and there were roughly 480,000 opioid-related deaths in 2019.^1,2^ OUD prevalence is higher among individuals with psychiatric disorders, chronic pain, and socio-economic deprivation.^3^

Medications for opioid use disorder (MOUD), such as methadone and buprenorphine, are licensed in many jurisdictions. They act on opioid receptors to reduce cravings and withdrawal symptoms. MOUD is evidence-based for reducing opioid dependence and harms including blood-borne virus infection and drug death.^3,4^ MOUD is included in the WHO Model List of Essential Medicines.^5^

A significant treatment gap exists. Globally, around 18–22% of people who inject opioids receive MOUD, with the gap widest in Africa, Asia, and rural settings.^6^

### Telemedicine

Telemedicine (TM) employs information and communication technologies to deliver healthcare services when patients and providers are geographically separated. TM adoption for general healthcare expanded rapidly in many countries during and after the COVID-19 pandemic.

TM offers significant benefits by addressing barriers to access and treatment engagement.^7,8^ It reduces waiting times and expands care delivery to community settings. TM can improve service efficiency and reduce costs by decreasing non-attendance, minimising administrative burdens, optimising workforce distribution, and reducing the need for physical infrastructure.^9,10^ Improved patient retention, continuity of care, and treatment adherence improve outcomes and reduce costs. TM also benefits socially excluded or isolated populations facing barriers to engagement, including those in underserved rural and geographically remote areas.^11–13^

The distribution and maturity of TM vary globally, with more widespread adoption and advanced development in North America, Europe, and other high-income regions. The scientific literature reflects these trends, although emerging ‘grey literature’ evidence exists in other languages and regions.^14,15^

Persistent infrastructural, socio-technical, and regulatory barriers limit the provision, accessibility, and outcomes of TM.^7^ A digital divide, particularly in resource-limited settings, hinders service reach.^13^ The pandemic-driven expansion of TM exposed further challenges including fragmented integration, reimbursement uncertainties, privacy concerns, diagnostic limitations, and perceived rapport erosion, affecting acceptability.^8,16^ Addressing these interconnected barriers through development of infrastructure, access to connected devices, provider and patient training, regulatory frameworks, and clinical guidance is crucial to realise the potential of TM.

### Telemedicine-enabled medication for OUD

Telemedicine to support MOUD (TMOUD) has emerged as a critical tool to expand care for people with OUD, many of whom face barriers to treatment, and there is growing evidence of its acceptability and effectiveness.^8,^^17–24^

A network of systemic barriers limits TMOUD's potential. Healthcare policy, regulation, and governance frameworks define rules and standards for care. TMOUD delivery can be impeded by policies concerning patient privacy, prescription management, and reimbursement for consultations.^25–28^ Regulations governing controlled substances like methadone and buprenorphine are a specific issue.^28^ A disconnect between evolving healthcare delivery models and professional guidelines creates a barrier for services seeking to implement TMOUD.^26,27,29^ Limited evidence of TMOUD effectiveness, safety, and quality hinders policy and guidance development.^17^

Inadequate digital infrastructure also hinders equitable TMOUD provision.^30^ Providers and patients in remote areas may experience coverage gaps due to unreliable or unaffordable internet. People with OUD are often from marginalised communities, including disadvantaged ethnic groups, displaced people, and those experiencing socio-economic exclusion, lower incomes, higher rates of (digital) illiteracy, multi-morbidity, and precarity from imprisonment and homelessness. Those who might benefit most may be excluded if they lack the skills or resources to utilise the necessary technology, for example, smartphones/computers, and multi-layered authentication, and service-mandated videoconferencing platforms.^20,31^

Healthcare providers may be reluctant to implement TMOUD due to concerns about consultation quality, including the lack of physical examination and toxicology screening.^16,17,31^ Barriers include the lack of electronic health records and the inability to transmit prescriptions to pharmacies.^20,32^ Clinicians may also lack the skills, experience, and professional guidance to design and deliver remote care models. Providers may have concerns about resource implications, including workload and reimbursement for remote consultations.^16,26,32,33^

There is a striking difference between high-income countries (HICs) and low- and middle-income countries (LMICs) in TMOUD use. Barriers in LMICs include a lack of awareness, technology, and resources, digital literacy, and programme, organisational, regulatory, and funding limitations.^13,34^ Critically, there is a lack of LMIC-relevant research and the perspectives of addiction care professionals and patients on TMOUD use, barriers, and facilitators are lacking.

### World addiction medicine reports

The International Society of Addiction Medicine (ISAM) is a network of physicians with over 15,000 members from almost 100 countries. ISAM established the Global Experts Network (ISAM-GEN) to collate expert opinion and support consensus in addiction science. ISAM-GEN's initiatives include international longitudinal surveys examining trends in addiction policies, practices, and patterns of need. By capturing expert consensus and regional variations, these reports inform policy recommendations and clinical guidelines tailored to different contexts. ISAM-GEN's findings are expected to inform education and training priorities for addiction professionals worldwide.^35^ ISAM-GEN draws on a critical mass of addiction medicine experts from across the globe, providing comprehensive perspectives that transcend regional boundaries.

While there is a growing body of research on TM for OUD, most studies are either country-specific (predominantly the United States, Canada, and Europe) or limited to high-income contexts. Evidence from LMICs remains sparse, and few studies have compared priorities, practices, and barriers across diverse settings. Moreover, existing reviews are largely descriptive or focus on regulatory flexibilities during COVID-19, rather than providing global comparative perspectives from frontline addiction medicine experts.

The current study addresses these gaps by engaging an international network of addiction medicine experts across 37 countries. By directly comparing perspectives between high- and low-income settings, this work provides new insights into how structural, regulatory, and infrastructural factors shape the feasibility of TMOUD worldwide. These findings contribute unique, practice-based evidence to inform international policy, guidelines, and future implementation research.

## Methods

### Survey development

An advisory committee of 21 international experts was convened (Figure S1), including representation from 16 countries in all seven World Bank regions. Nine were from LMICs.

JTWT and JS designed a structured survey instrument, informed by international literature on factors associated with TM and TMOUD implementation. The advisory committee provided feedback. The final version (Supplementary materials) included sections on participant characteristics; current use of TM and TMOUD; national priorities relating to TMOUD; and barriers to its implementation and was created as an online survey using the Qualtrics platform.^36^

The School of Medicine Ethics Committee at the University of St Andrews, Scotland, approved the study (reference MD17377).

### Measures

Dichotomous (‘yes/no’) questions with a neutral ‘don't know’ option were used for current use and priorities for TMOUD. Perceptions of barriers were assessed using a 5-point Likert scale ('strongly agree’ to ‘strongly disagree’, with an ‘undecided’ option). Free-text questions allowed participants to elaborate.

TMOUD was defined as including assessment, treatment initiation/induction, and/or review of patients with OUD.

### Procedure

The survey was distributed between July and November 2024 to more than 250,000 members of the World Psychiatric Association, approximately 400 members of ISAM-GEN, and 34 other international experts in our network. Potential participants received an email invitation containing a study description, participant information sheet, eligibility criteria, and a link to the online questionnaire. Participation was voluntary and contingent on provision of informed consent, confirmed at the start of the online survey. Eligible participants were clinicians involved in OUD care or individuals in clinical or policy leadership roles. Three reminder emails encouraging participation were circulated approximately monthly.

### Analysis

Responses were anonymised before analysis in R Studio. Responses were grouped: for dichotomous questions, ‘don't know’ and missing responses were combined; Likert responses were collapsed into ‘Strongly agree/agree’, ‘Undecided’, and ‘Disagree/strongly disagree’. Countries were coded as low/middle-income (LMIC) or high-income (HIC) using the World Bank classification. Frequencies and percentages were reported in tables and figures organised by country income group and current TMOUD provision.

## Results

### Participant and country/jurisdiction characteristics

**Figure 1.**
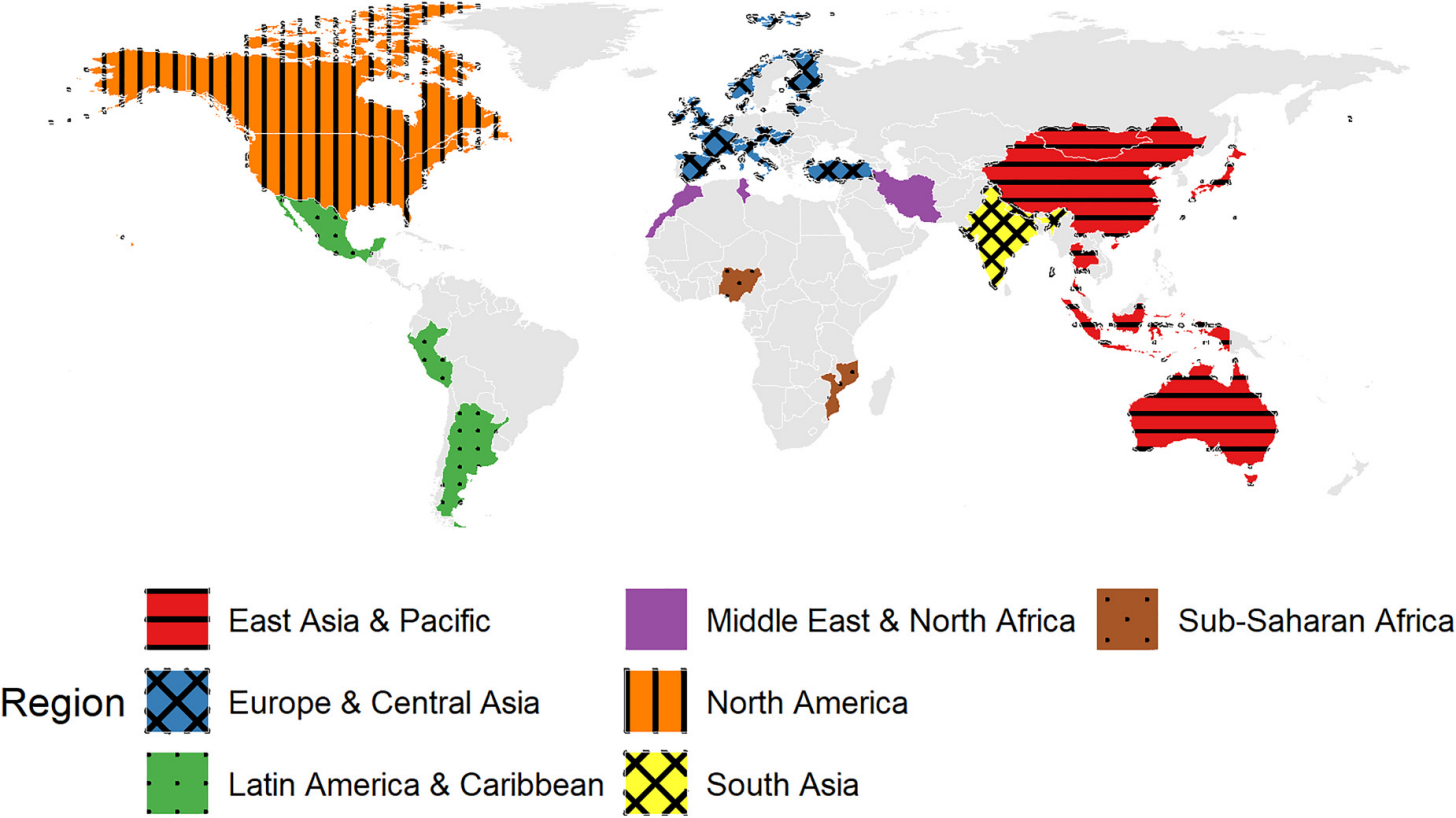
Geographical distribution of participants by country and World Bank region.

Sixty-eight participants from 37 countries, representing all seven World Bank regions, submitted responses (Figure 1, Table S1). Responses were received from the Europe and Central Asia (46%), East Asia and Pacific (15%), and South Asia (12%) regions. Almost one-third (32%) were from LMICs.

Most participants (60%) were aged 26–50 years, and just over one-third (35%) were female (Table 1). Participants generally had multiple roles in addiction treatment (median (interquartile range), 2 (1, 4)), predominantly as clinical academics (66%) and/or addiction clinicians (60%). Respondents included those with a clinical leadership role (41%) and/or researchers and scientists (38%).

**Table 1.** Participant characteristics.

	Number	Percentage
Age group		
26–50	41	60
50–65	24	35
Over 65	3	4
Gender		
Female	24	35
Male	43	63
Prefer not to say	1	1
Area of activity in relation to the addiction treatment sector		
Clinician	41	60
Clinical leadership	28	41
Clinical policymaker	13	19
Government advisor	9	13
Clinical academic	45	66
Researcher/scientist	26	38
Other, please specify	1	1

Just over a third (38%) reported that TMOUD was available in their country or jurisdiction at the time of participation (Table 2). This was markedly higher among HIC 58% (23/40) than LMIC 11% (3/28) participants.

**Table 2.** TMOUD availability by country income group.

	Low- and middle-income countries	High-income countries	Total
No current TMOUD	25	17	42
Current TMOUD	3	23	26
Total	28	40	68

TMOUD: telemedicine facilitated medication for opioid use disorder.

### Current use of TM for general healthcare

**Figure 2.**
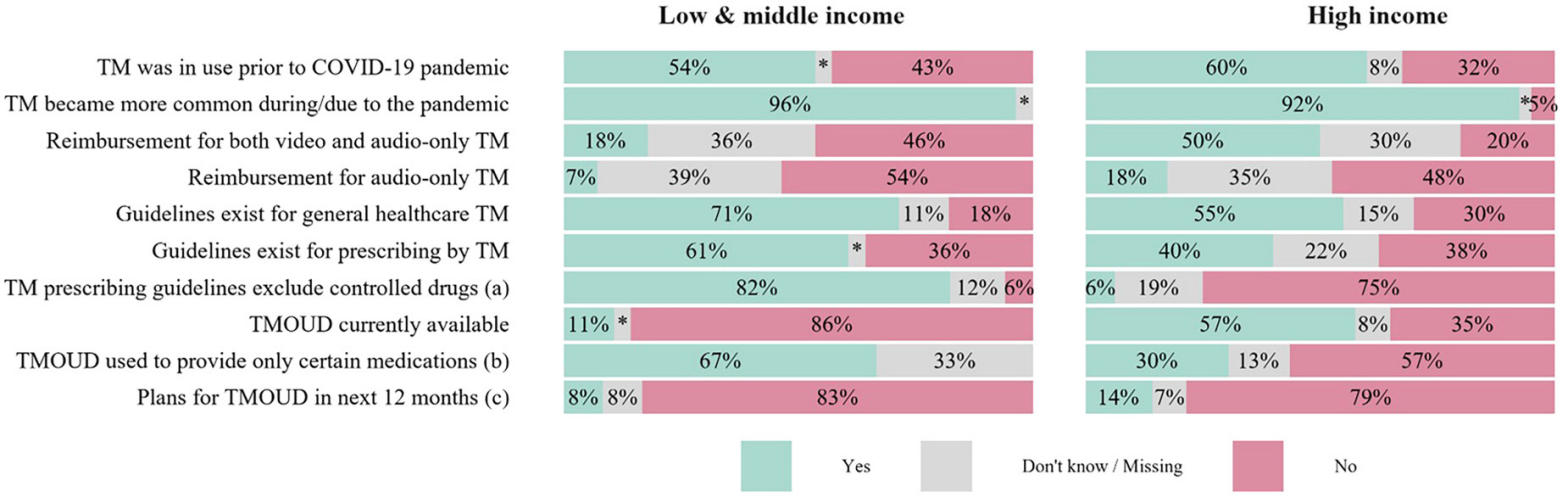
Current use of TM by country income group. TM: telemedicine; TMOUD: telemedicine to support medications for opioid use disorder. * denotes <5%; (a) among those indicating TM prescribing guidelines exist; (b) among those reporting TMOUD is currently available; (c) among those reporting TMOUD is not currently available.

Participants provided information on current TM arrangements (Figure 2, Table S2). Pre-COVID use of TM for general healthcare was reported by 57% of participants overall, with similar proportions across income groups and a higher proportion from areas currently providing TMOUD.

The pandemic substantially accelerated TM use, reported by 94%. This was relatively consistent across countries. Over half (62%) indicated that general healthcare TM guidelines were available, more commonly reported by participants from LMICs and from areas with current TMOUD. Guidance on remote prescribing was less frequently reported overall (49%), again more common in LMICs and TMOUD-experienced areas.

Where TM prescribing guidelines were available (*n* = 33), different allowances for controlled drug prescribing were identified. Overall, 45% reported these drugs were excluded from TM prescribing guidance, notably higher among those from LMICs and only reported by one HIC participant. Those from areas currently providing TMOUD were less likely to report such restrictions.

Overall, 37% of participants reported full reimbursement for both video and audio-only TM services, again notably higher among HICs and areas with current TMOUD. More LMIC participants reported a lack of payment for audio-only consultations.

### Current TMOUD provision

Overall, 38% of participants reported TMOUD was available in their jurisdiction, substantially higher among HIC participants. Among those reporting current TMOUD, 35% indicated this was only being used to provide certain medications, and 15% were unsure about prescribing restrictions. Limitations on the type of drugs that could be prescribed were more frequent in LMICs. In free-text responses, seven participants reported TMOUD prescribing was generally limited to methadone and/or buprenorphine, with no difference by country income group. One LMIC participant indicated some benzodiazepines and naltrexone were allowed.

In areas without current TMOUD, just 10% indicated plans to introduce services in the next 12 months. Fourteen percent either did not know or did not respond.

### TMOUD priorities

**Figure 3.**
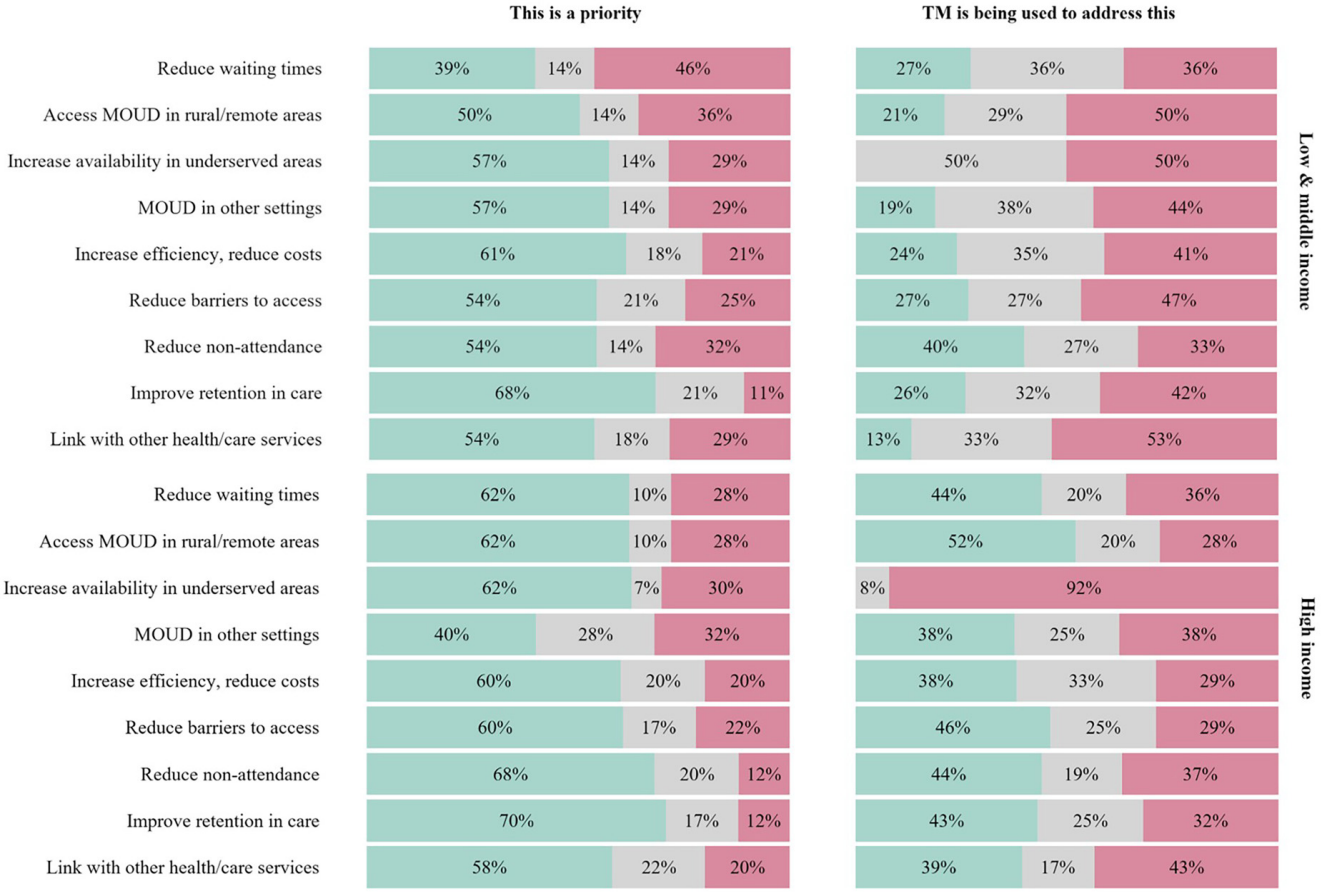
Priorities for TM for delivery of medications for treatment of opioid use disorder by country income group. TM: telemedicine; MOUD: medications for opioid use disorder.

Participants indicated which of nine statements describing potential TMOUD benefits were a high priority in their country or jurisdiction (Figure 3, Table S3). Overall, the most frequently endorsed items were: improving retention in treatment (69% overall), reducing non-attendance (62%), increasing MOUD availability in underserved (60%) or rural and geographically remote (57%) areas, and increasing service efficiency and reducing costs (60%). Reducing waiting times to access MOUD and making MOUD available in other service settings received lower support (53% and 47%, respectively).

Endorsement rates among respondents from HICs were higher than those from LMICs for reducing waiting times and expanding MOUD accessibility to rural and geographically remote areas. A larger share of LMIC respondents prioritised offering MOUD in other service settings. Both groups indicated similar support for increasing MOUD availability in underserved areas and improving retention in care.

For each statement of potential benefits, participants who indicated this was a high priority also indicated if TMOUD was being used to address the issue.

Overall, HIC respondents more frequently reported that TM was being used to meet high-priority goals than those from LMICs. For instance, among individuals who rated reducing waiting times as a high priority, 39% overall reported TMOUD was being used to address this, predominantly those from HICs. Similar disparities were observed for reports of whether TMOUD implementation included improving retention in treatment (36% overall) and reducing barriers to access (38% overall). Although LMIC participants often endorsed many of the same priorities, they were less likely to confirm TMOUD was being used to address these issues and a greater proportion replied ‘don't know’ or did not respond, suggesting a disconnect between priorities and practices.

### Barriers to TMOUD

#### Policy and guidance

**Figure 4.**
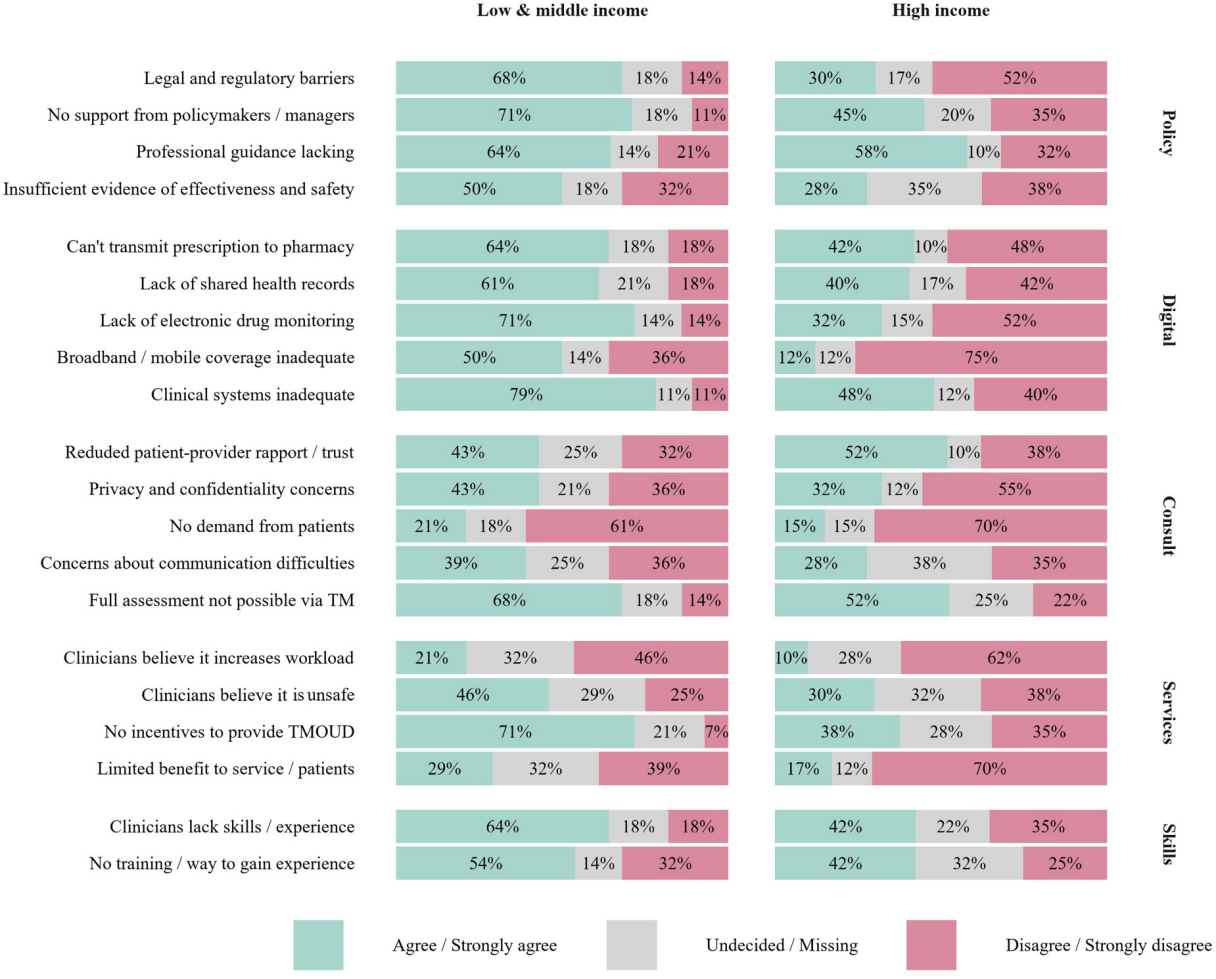
Barriers to provision of TM for treatment of opioid use disorder by country income group. TM: telemedicine; (TM)OUD: (telemedicine for treatment of) opioid use disorder.

Overall, 60% of respondents agreed that TMOUD was not prioritised or was unsupported by policymakers or managers, with more endorsement from LMICs (Figure 4, Table S4). Most agreed there was insufficient guidance from their professional organisation on providing TMOUD, again higher among LMICs and areas not currently providing TMOUD.

Almost half (48%) agreed there were too many legal or regulatory barriers. Participants from HICs and jurisdictions providing TMOUD were approximately half as likely to report this.

#### Digital infrastructure

Over one-quarter of respondents (29%) agreed that broadband and/or mobile coverage was inadequate. This barrier was strongly associated with lower-income countries and areas not providing TMOUD.

Several challenges with clinical systems were noted. Overall, 63% agreed that current clinical systems were inadequate. Just over half (52%) agreed that the lack of shared electronic health records was a barrier, with a marked difference by country income, but none by current TMOUD provision.

The lack of electronic transmission of MOUD prescriptions from prescriber to pharmacy was a challenge for 56% of participants overall. Around half (52%) agreed the lack of an electronic drug monitoring system was a barrier. These barriers were more apparent in LMICs than HICs.

#### Health/clinical systems

A minority (23%) agreed that TMOUD offered limited benefit, and just over half (56%) reported there were no incentives to provide it. There was broad agreement that patients would welcome TMOUD, and few participants (18%) agreed there was no demand for it from patients. Perceptions of patient demand were relatively consistent across country income groups and by current TMOUD provision.

#### Clinician perspectives

Just 15% agreed that clinicians believed TMOUD increased their workload, again higher in LMICs but relatively consistent by current TMOUD status. Overall, 38% believed TMOUD was clinically unsafe, more frequently endorsed among those from LMICs and areas without TMOUD.

While just over half (54%) agreed clinicians lacked the necessary experience or training, the proportion decreased with country income and TMOUD experience. Similar proportions and disparities were observed for the statement ‘there is no training or way to gain the necessary experience for clinicians’.

#### Healthcare consultations

There was general support for the statement ‘clinicians believe it is not possible to complete a full assessment without an in-person appointment’ (62% overall). Around half (52%) agreed that reduced patient–provider rapport and trust was a barrier, and this was relatively consistent across income group and by current TMOUD status.

Less than half (34%) endorsed concerns about communication difficulties, slightly higher among those from LMICs but little difference by current TMOUD status. Overall, 38% agreed there were concerns over privacy and confidentiality.

#### Evidence base

Overall, 38% of participants agreed there was insufficient evidence of TMOUD effectiveness and safety. While this was less frequent among participants from HICs and areas providing TMOUD, there was more uncertainty about the evidence base (higher proportion of ‘Undecided/missing’ responses) from LMICs and jurisdictions without TMOUD.

## Discussion

This is the first study to map global practices, priorities, and barriers to TMOUD from the perspective of addiction medicine experts, providing data from both high- and low-income contexts. Unlike previous studies, which have mainly reported evidence from single countries generally in high-income regions, our findings highlight cross-national disparities and identify specific gaps in LMIC infrastructure, policy support, and clinician training. By exploring TMOUD within a comparative global framework, this study generates actionable evidence to guide equitable implementation strategies that would not be apparent from country-level analyses alone.

### Participant characteristics

Findings from 68 experts across 37 countries highlight global diversity in perceptions and experiences of TMOUD. While most respondents were from Europe, Central Asia, and higher-income nations, important perspectives emerged from LMICs.

### Current use of TM for general healthcare

TM was well established in many countries pre-COVID-19, and the pandemic significantly accelerated its adoption. TM has been widely developed to enable MOUD. However, disparities persist: many countries have yet to implement guidelines for remote MOUD prescribing, and reimbursement for TM remains uneven, especially in LMICs. It is notable that 61% of participants from areas providing TMOUD reported parity of reimbursement for both video and audio-only consultations, compared with only 31% from areas without TMOUD. This suggests that reimbursement structures may play a role in shaping TMOUD availability and uptake. Countries that do not provide equivalent reimbursement for different TM modalities risk exacerbating inequities in access, particularly for populations with lower digital literacy, limited digital infrastructure, or financial barriers to video consultations.^14,15^

### Current TMOUD provision

Only about one-third of participants, predominantly in HICs, reported that TMOUD was in place in their jurisdiction. This was unsurprising, considering that HICs were better prepared to implement TM due to pre-existing digital infrastructures, resource availability, and supportive policy contexts.^37^ Yet, it also highlights the critical challenge of scaling up TMOUD in LMICs, where infrastructure deficits, regulatory complexity, and budgetary constraints can be challenges.^34,38^ There was minimal evidence of planned TMOUD expansion in regions where it is not currently available, irrespective of income group, suggesting that implementation may have stagnated.

### TMOUD priorities

Respondents identified improving retention, expanding access, and enhancing efficiency as top priorities, aligning with benefits in the broader TM literature. However, differences by income group (e.g. LMIC respondents prioritising delivery of MOUD in alternative settings, HIC respondents focusing on reducing wait times) suggest variability in TMOUD drivers across countries. TMOUD expansion efforts must recognise and respond to significant contextual variability. There may also be a lack of research on innovative practices in LMICs, reducing awareness of the potential for TMOUD to address treatment gap issues.^34,39^

### Barriers to TMOUD

A major theme was the perception that TMOUD was insufficiently supported by policymakers, especially in LMICs, and that guidance and standards for remote prescribing were inadequate. Regulatory complexities, particularly concerning controlled medications, continue to hamper TMOUD expansion.^40–42^ In many settings, especially LMICs, the lack of digital infrastructure, integrated electronic health records, and remote prescribing are substantial barriers.^34,39,42^ A significant proportion believed that clinicians needed more training and support, and that professional development opportunities are insufficient.

A knowledge translation gap may contribute to hesitancy in adopting TMOUD, particularly in LMICs and areas currently lacking services.^13,42^ While there is evidence of TMOUD effectiveness and safety, around half of LMIC participants and those from non-TMOUD areas believe this to be insufficient. The generation and exchange of learning from regionally relevant research is required to bridge this gap.^34^

### Strengths and weaknesses

This is the first global survey to examine the experiences and perceptions of addiction medicine experts on using TM to deliver treatment for people with OUD. Responses were received from all regions of the world. However, limited representation of some world regions, especially African countries, underscores the continued need for broader global engagement, particularly in under-resourced areas that bear the greatest burden of untreated OUD.

Survey invitations were disseminated to the general membership of the World Psychiatric Association and ISAM-GEN; however, the proportion of recipients who met the study's eligibility criteria was unknown. In the absence of a unified distribution list and an eligible denominator, the response rate could not be calculated.

Self-selection may have skewed results to those with an interest in or experience of TM(OUD). Most countries were represented by one or two participants, but larger numbers of responses were received from India (7) and the UK (11). While they could represent sub-national jurisdictions with distinct TM(OUD) policies and practices, their data may represent inconsistent and individual perceptions of priorities and barriers.

### Overall implications and conclusions

This study provides the first global examination of perceptions and experiences of TMOUD, highlighting significant disparities across HICs and LMICs. While TM has the potential to expand access, its implementation remains uneven due to regulatory, infrastructural, and clinical challenges.

Findings indicate TMOUD is more widely available in HICs, where digital infrastructure, clinical guidelines, and reimbursement mechanisms are generally more developed. In contrast, professionals in LMICs face more systemic barriers, including limited digital infrastructure, prescribing regulations, and insufficient training. Importantly, policymakers in LMICs were perceived as less supportive, underscoring the need for targeted advocacy and policy reform.

To advance equitable global implementation, three key priorities emerge:

1. Developing and standardising clinical guidance: Countries should establish clear, evidence-based TMOUD guidelines, particularly for prescribing, aligned with international best practices and evidence while allowing for regional and cultural adaptations.
2. Investing in digital health infrastructure: Bridging the digital divide, particularly in LMICs, is critical. This includes improving information and communication technology equipment and internet connectivity, electronic health record systems, and e-prescribing platforms.
3. Strengthening workforce capacity and clinician training: Many healthcare providers lack experience with TM for addiction care. Expanding training programmes, particularly in LMICs, will be essential.

Future research should prioritise rigorous evaluation of TMOUD models outside high-income settings, particularly in non-English-speaking regions and LMICs where current evidence is sparse. Comparative studies assessing clinical outcomes, cost-effectiveness, and patient acceptability across different regulatory and healthcare environments will be crucial for informing global best practices. There is a pressing need to share evidence of TMOUD safety and effectiveness, and to translate this knowledge across regions to support implementation in different contexts.

Given the scale of the opioid crisis, the stark treatment gap, and the barriers to in-person care, TM represents a transformative yet underutilised strategy for improving OUD treatment access worldwide. International collaboration, policy reform, and strategic investment are essential to fully realise the potential of TMOUD and ensure equitable access.

## Supplemental Material

sj-docx-1-jtt-10.1177_1357633X251394442 - Supplemental material for Global perspectives on telemedicine-enabled medications for opioid use disorder: Practices, priorities, and barriers Supplemental material, sj-docx-1-jtt-10.1177_1357633X251394442 for Global perspectives on telemedicine-enabled medications for opioid use disorder: Practices, priorities, and barriers by Joe Schofield, Alexander Mario Baldacchino, Atul Ambekar, Honest Anaba, Jenna L Butner, Nathaniel Day, Hamed Ekhtiari, Fatima Elomari, Marica Ferri, Konstantinos Kokkolis, Christos Kouimtsidis, Jonna Levola, Jiang Long, David Martell, Dario Gigena Parker, Afarin Rahimi-Movaghar, Kristiana Siste, Scott Steiger, Arash Khojasteh Zonoozi and Joseph Tay Wee Teck in Journal of Telemedicine and Telecare
