# Supplementary material for: Global perspectives on telemedicine-enabled medications for opioid use disorder: Practices, priorities, and barriers
Source: J Telemed Telecare. 2026 Jan 21;32(7):601–11. doi: 10.1177/1357633X251394442 (PMC13421156; doi:10.1177/1357633X251394442)

**Supplementary material**

**Contents**

[Figure S1 Advisory group 1](#_Toc197937836)

[Table S1 Responses by country, region, and income group 1](#_Toc197937837)

[Table S2 Current use of telemedicine 3](#_Toc197937838)

[Survey instrument 7](#_Toc197937839)


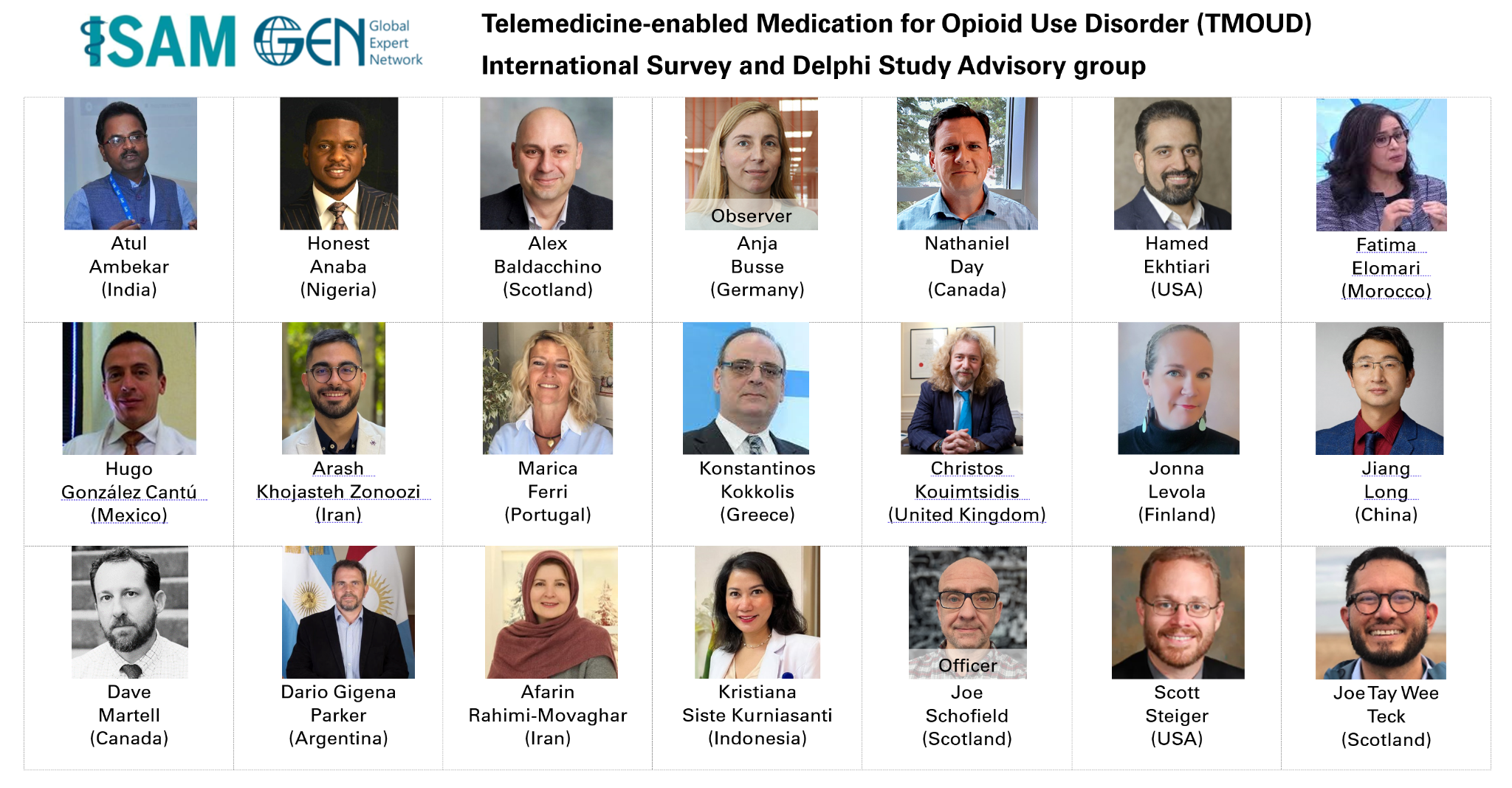


# Figure S1 Advisory group

# Table S1 Responses by country, region, and income group

| **Country** | **World Bank region** | **World Bank income group** | **Responses** |
| --- | --- | --- | --- |
| Argentina | Latin America & Caribbean | Low to middle | 1 |
| Australia | East Asia & Pacific | High | 2 |
| Austria | Europe & Central Asia | High | 1 |
| Canada | North America | High | 4 |
| China | East Asia & Pacific | Low to middle | 2 |
| Czech Republic | Europe & Central Asia | High | 1 |
| Denmark | Europe & Central Asia | High | 1 |
| Estonia | Europe & Central Asia | High | 1 |
| Finland | Europe & Central Asia | High | 2 |
| France | Europe & Central Asia | High | 1 |
| Hungary | Europe & Central Asia | High | 1 |
| India | South Asia | Low to middle | 7 |
| Indonesia | East Asia & Pacific | Low to middle | 3 |
| Iran | Middle East & North Africa | Low to middle | 1 |
| Ireland | Europe & Central Asia | High | 2 |
| Italy | Europe & Central Asia | High | 1 |
| Japan | East Asia & Pacific | High | 1 |
| Lithuania | Europe & Central Asia | High | 1 |
| Malta | Europe & Central Asia | High | 1 |
| Mexico | Latin America & Caribbean | Low to middle | 2 |
| Mongolia | East Asia & Pacific | Low to middle | 1 |
| Morocco | Middle East & North Africa | Low to middle | 1 |
| Mozambique | Sub-Saharan Africa | Low to middle | 1 |
| Nepal | South Asia | Low to middle | 1 |
| Netherlands | Europe & Central Asia | High | 1 |
| Nigeria | Sub-Saharan Africa | Low to middle | 4 |
| Norway | Europe & Central Asia | High | 1 |
| Peru | Latin America & Caribbean | Low to middle | 1 |
| Slovakia | Europe & Central Asia | High | 1 |
| Slovenia | Europe & Central Asia | High | 1 |
| Spain | Europe & Central Asia | High | 2 |
| Switzerland | Europe & Central Asia | High | 1 |
| Thailand | East Asia & Pacific | Low to middle | 1 |
| Tunisia | Middle East & North Africa | Low to middle | 1 |
| Turkey | Europe & Central Asia | Low to middle | 1 |
| UK | Europe & Central Asia | High | 11 |
| USA | North America | High | 2 |

# Table S2 Current use of telemedicine

|  |  | **By country income group** | | | | | | |  |  | **By current TMOUD** | | | | | | |
| --- | --- | --- | --- | --- | --- | --- | --- | --- | --- | --- | --- | --- | --- | --- | --- | --- | --- |
|  | **Country income** | **Yes** | | **DK /  missing** | | **No** | | **Total** |  | **Current TMOUD** | **Yes** | | **DK /  missing** | | **No** | | **Total** |
| **In general, TM was already in use in my jurisdiction prior to the COVID-19 pandemic** | High | 24 | 60% | 3 | 8% | 13 | 33% | 40 |  | No | 19 | 45% | 2 | 5% | 21 | 50% | 42 |
|  | Low-Mid | 15 | 54% | 1 | 4% | 12 | 43% | 28 |  | Yes | 20 | 77% | 2 | 8% | 4 | 15% | 26 |
|  | **All** | **39** | **57%** | **4** | **6%** | **25** | **37%** | **68** |  | **All** | **39** | **57%** | **4** | **6%** | **25** | **37%** | **68** |
| **In general, TM became more common as a result of or during the COVID-19 pandemic** | High | 37 | 93% | 1 | 3% | 2 | 5% | 40 |  | No | 38 | 90% | 2 | 5% | 2 | 5% | 42 |
|  | Low-Mid | 27 | 96% | 1 | 4% | 0 | 0% | 28 |  | Yes | 26 | 100% | 0 | 0% | 0 | 0% | 26 |
|  | **All** | **64** | **94%** | **2** | **3%** | **2** | **3%** | **68** |  | **All** | **64** | **94%** | **2** | **3%** | **2** | **3%** | **68** |
| **There are guidelines on the use of TM to provide general healthcare** | High | 22 | 55% | 6 | 15% | 12 | 30% | 40 |  | No | 21 | 50% | 8 | 19% | 13 | 31% | 42 |
|  | Low-Mid | 20 | 71% | 3 | 11% | 5 | 18% | 28 |  | Yes | 21 | 81% | 1 | 4% | 4 | 15% | 26 |
|  | **All** | **42** | **62%** | **9** | **13%** | **17** | **25%** | **68** |  | **All** | **42** | **62%** | **9** | **13%** | **17** | **25%** | **68** |
| **In general, there are guidelines on the use of TM to prescribe medications** | High | 16 | 40% | 9 | 23% | 15 | 38% | 40 |  | No | 17 | 40% | 6 | 14% | 19 | 45% | 42 |
|  | Low-Mid | 17 | 61% | 1 | 4% | 10 | 36% | 28 |  | Yes | 16 | 62% | 4 | 15% | 6 | 23% | 26 |
|  | **All** | **33** | **49%** | **10** | **15%** | **25** | **37%** | **68** |  | **All** | **33** | **49%** | **10** | **15%** | **25** | **37%** | **68** |
| **Current guidelines to prescribe medications via TM specifically excludes controlled drugs** (among those with prescribing guidelines) | High | 1 | 6% | 12 | 75% | 3 | 19% | 16 |  | No | 12 | 71% | 3 | 18% | 2 | 12% | 17 |
|  | Low-Mid | 14 | 82% | 1 | 6% | 2 | 12% | 17 |  | Yes | 3 | 19% | 2 | 13% | 11 | 69% | 16 |
|  | **All** | **15** | **45%** | **13** | **39%** | **5** | **15%** | **33** |  | **All** | **15** | **45%** | **5** | **15%** | **13** | **39%** | **33** |
| **Services are fully reimbursed for providing both video and audio-only telemedicine services** | High | 20 | 50% | 12 | 30% | 8 | 20% | 40 |  | No | 11 | 31% | 9 | 25% | 16 | 44% | 36 |
|  | Low-Mid | 5 | 18% | 10 | 36% | 13 | 46% | 28 |  | Yes | 14 | 61% | 4 | 17% | 5 | 22% | 23 |
|  | **All** | **25** | **37%** | **22** | **32%** | **21** | **31%** | **68** |  | **All** | **25** | **42%** | **13** | **22%** | **21** | **36%** | **59** |
| **Services are fully reimbursed only for video consults. Services are not reimbursed for audio-only consults.** | High | 7 | 18% | 14 | 35% | 19 | 48% | 40 |  | No | 4 | 11% | 10 | 28% | 22 | 61% | 36 |
|  | Low-Mid | 2 | 7% | 11 | 39% | 15 | 54% | 28 |  | Yes | 5 | 24% | 4 | 19% | 12 | 57% | 21 |
|  | **All** | **9** | **13%** | **25** | **37%** | **34** | **50%** | **68** |  | **All** | **9** | **16%** | **14** | **25%** | **34** | **60%** | **57** |
| **Telemedicine is currently being used to enable the provision of MOUD** | High | 23 | 58% | 3 | 8% | 14 | 35% | 40 |  | No | 0 | 0% | 2 | 5% | 40 | 95% | 42 |
|  | Low-Mid | 3 | 11% | 1 | 4% | 24 | 86% | 28 |  | Yes | 26 | 100% | 0 | 0% | 0 | 0% | 26 |
|  | **All** | **26** | **38%** | **4** | **6%** | **38** | **56%** | **68** |  | **All** | **26** | **38%** | **2** | **3%** | **40** | **59%** | **68** |
| **Telemedicine is currently being used to provide only certain MOUD** (among those with current TMOUD) | High | 7 | 30% | 3 | 13% | 13 | 57% | 23 |  | No |  |  |  |  |  |  |  |
|  | Low-Mid | 2 | 67% | 1 | 33% | 0 | 0% | 3 |  | Yes | 9 | 35% | 4 | 15% | 13 | 50% | 26 |
|  | **All** | **9** | **35%** | **4** | **15%** | **13** | **50%** | **26** |  | **All** |  |  |  |  |  |  |  |
| **My jurisdiction is planning to use TM to enable the provision of MOUD in the next 12 months** (among those saying no current TMOUD | High | 2 | 12% | 3 | 18% | 12 | 71% | 17 |  | No | 4 | 10% | 6 | 14% | 32 | 76% | 42 |
|  | Low-Mid | 2 | 8% | 3 | 12% | 20 | 80% | 25 |  | Yes |  |  |  |  |  |  |  |
|  | **All** | **4** | **10%** | **6** | **14%** | **32** | **76%** | **42** |  | **All** |  |  |  |  |  |  |  |

**Table S3 Priorities for TMOUD**

|  |  | **This statement is a high priority in my jurisdiction** | | | | | | | | | | | | | | | |
| --- | --- | --- | --- | --- | --- | --- | --- | --- | --- | --- | --- | --- | --- | --- | --- | --- | --- |
|  |  | **By country income group** | | | | | | |  |  | **By current TMOUD** | | | | | | |
|  | **Country income** | **Yes** | | **DK/missing** | | **No** | | **Total** |  | **Current TMOUD** | **Yes** | | **DK/missing** | | **No** | | **Total** |
| **Reduce waiting times to access MOUD** | High | 25 | 63% | 4 | 10% | 11 | 28% | **40** |  | No | 15 | 36% | 7 | 17% | 20 | 48% | **42** |
|  | Low-Mid | 11 | 39% | 4 | 14% | 13 | 46% | **28** |  | Yes | 21 | 81% | 1 | 4% | 4 | 15% | **26** |
|  | **All** | **36** | **53%** | **8** | **12%** | **24** | **35%** | **68** |  | **All** | **36** | **53%** | **8** | **12%** | **24** | **35%** | **68** |
| **MOUD accessible to rural and geographically remote areas** | High | 25 | 63% | 4 | 10% | 11 | 28% | **40** |  | No | 18 | 43% | 7 | 17% | 17 | 40% | **42** |
|  | Low-Mid | 14 | 50% | 4 | 14% | 10 | 36% | **28** |  | Yes | 21 | 81% | 1 | 4% | 4 | 15% | **26** |
|  | **All** | **39** | **57%** | **8** | **12%** | **21** | **31%** | **68** |  | **All** | **39** | **57%** | **8** | **12%** | **21** | **31%** | **68** |
| **Increasing MOUD availability in underserved areas** | High | 25 | 63% | 3 | 8% | 12 | 30% | **40** |  | No | 21 | 50% | 6 | 14% | 15 | 36% | **42** |
|  | Low-Mid | 16 | 57% | 4 | 14% | 8 | 29% | **28** |  | Yes | 20 | 77% | 1 | 4% | 5 | 19% | **26** |
|  | **All** | **41** | **60%** | **7** | **10%** | **20** | **29%** | **68** |  | **All** | **41** | **60%** | **7** | **10%** | **20** | **29%** | **68** |
| **MOUD in other service settings** | High | 16 | 40% | 11 | 28% | 13 | 33% | **40** |  | No | 20 | 48% | 8 | 19% | 14 | 33% | **42** |
|  | Low-Mid | 16 | 57% | 4 | 14% | 8 | 29% | **28** |  | Yes | 12 | 46% | 7 | 27% | 7 | 27% | **26** |
|  | **All** | **32** | **47%** | **15** | **22%** | **21** | **31%** | **68** |  | **All** | **32** | **47%** | **15** | **22%** | **21** | **31%** | **68** |
| **Increase service efficiency and reduce costs** | High | 24 | 60% | 8 | 20% | 8 | 20% | **40** |  | No | 23 | 55% | 8 | 19% | 11 | 26% | **42** |
|  | Low-Mid | 17 | 61% | 5 | 18% | 6 | 21% | **28** |  | Yes | 18 | 69% | 5 | 19% | 3 | 12% | **26** |
|  | **All** | **41** | **60%** | **13** | **19%** | **14** | **21%** | **68** |  | **All** | **41** | **60%** | **13** | **19%** | **14** | **21%** | **68** |
| **Reduce barriers to access including opening hours, travel costs, gender-specific spaces** | High | 24 | 60% | 7 | 18% | 9 | 23% | **40** |  | No | 21 | 50% | 10 | 24% | 11 | 26% | **42** |
|  | Low-Mid | 15 | 54% | 6 | 21% | 7 | 25% | **28** |  | Yes | 18 | 69% | 3 | 12% | 5 | 19% | **26** |
|  | **All** | **39** | **57%** | **13** | **19%** | **16** | **24%** | **68** |  | **All** | **39** | **57%** | **13** | **19%** | **16** | **24%** | **68** |
| **Reduce non-attendance at MOUD clinic appointments** | High | 27 | 68% | 8 | 20% | 5 | 13% | **40** |  | No | 22 | 52% | 7 | 17% | 13 | 31% | **42** |
|  | Low-Mid | 15 | 54% | 4 | 14% | 9 | 32% | **28** |  | Yes | 20 | 77% | 5 | 19% | 1 | 4% | **26** |
|  | **All** | **42** | **62%** | **12** | **18%** | **14** | **21%** | **68** |  | **All** | **42** | **62%** | **12** | **18%** | **14** | **21%** | **68** |
| **Improve retention in treatment** | High | 28 | 70% | 7 | 18% | 5 | 13% | **40** |  | No | 26 | 62% | 9 | 21% | 7 | 17% | **42** |
|  | Low-Mid | 19 | 68% | 6 | 21% | 3 | 11% | **28** |  | Yes | 21 | 81% | 4 | 15% | 1 | 4% | **26** |
|  | **All** | **47** | **69%** | **13** | **19%** | **8** | **12%** | **68** |  | **All** | **47** | **69%** | **13** | **19%** | **8** | **12%** | **68** |
| **Link people with other health and social care needs and services** | High | 23 | 58% | 9 | 23% | 8 | 20% | **40** |  | No | 23 | 55% | 7 | 17% | 12 | 29% | **42** |
|  | Low-Mid | 15 | 54% | 5 | 18% | 8 | 29% | **28** |  | Yes | 15 | 58% | 7 | 27% | 4 | 15% | **26** |
|  | **All** | **38** | **56%** | **14** | **21%** | **16** | **24%** | **68** |  | **All** | **38** | **56%** | **14** | **21%** | **16** | **24%** | **68** |

**Table S4 Barriers to TMOUD**

|  |  |  | **By country income group** | | | | | | |  |  | **By current TMOUD** | | | | | | |
| --- | --- | --- | --- | --- | --- | --- | --- | --- | --- | --- | --- | --- | --- | --- | --- | --- | --- | --- |
| **Domain** | **Issue** | **Country income** | **Strongly agree / Agree** | | **Undecided** | | **Disagree / Strongly disagree** | | **Total** |  | **Current TMOUD** | **Strongly agree / Agree** | | **Undecided** | | **Disagree / Strongly disagree** | | **Total** |
| **Policy and guidance** | **It is not prioritised or is unsupported by policymakers or managers** | High | 18 | 47% | 6 | 16% | 14 | 37% | **38** |  | No | 27 | 64% | 9 | 21% | 6 | 14% | **42** |
|  |  | Low-Mid | 20 | 80% | 2 | 8% | 3 | 12% | **25** |  | Yes | 11 | 42% | 4 | 15% | 11 | 42% | **26** |
|  |  | **All** | **38** | **60%** | **8** | **13%** | **17** | **27%** | **63** |  | **All** | **38** | **56%** | **13** | **19%** | **17** | **25%** | **68** |
| **Policy and guidance** | **Insufficient guidance from my professional organization, service or medical union** | High | 23 | 58% | 4 | 10% | 13 | 33% | **40** |  | No | 29 | 69% | 7 | 17% | 6 | 14% | **42** |
|  |  | Low-Mid | 18 | 72% | 1 | 4% | 6 | 24% | **25** |  | Yes | 12 | 46% | 1 | 4% | 13 | 50% | **26** |
|  |  | **All** | **41** | **63%** | **5** | **8%** | **19** | **29%** | **65** |  | **All** | **41** | **60%** | **8** | **12%** | **19** | **28%** | **68** |
| **Policy and guidance** | **There are too many legal and regulatory barriers** | High | 12 | 30% | 7 | 18% | 21 | 53% | **40** |  | No | 24 | 57% | 12 | 29% | 6 | 14% | **42** |
|  |  | Low-Mid | 19 | 79% | 1 | 4% | 4 | 17% | **24** |  | Yes | 7 | 27% |  | 0% | 19 | 73% | **26** |
|  |  | **All** | **31** | **48%** | **8** | **13%** | **25** | **39%** | **64** |  | **All** | **31** | **46%** | **12** | **18%** | **25** | **37%** | **68** |
| **Digital infra-structure** | **Broadband / mobile coverage inadequate** | High | 5 | 13% | 5 | 13% | 30 | 75% | **40** |  | No | 14 | 33% | 5 | 12% | 23 | 55% | **42** |
|  |  | Low-Mid | 14 | 56% | 1 | 4% | 10 | 40% | **25** |  | Yes | 5 | 19% | 4 | 15% | 17 | 65% | **26** |
|  |  | **All** | **19** | **29%** | **6** | **9%** | **40** | **62%** | **65** |  | **All** | **19** | **28%** | **9** | **13%** | **40** | **59%** | **68** |
| **Digital infra-structure** | **Current clinical systems are inadequate to provide it** | High | 19 | 48% | 5 | 13% | 16 | 40% | **40** |  | No | 27 | 64% | 6 | 14% | 9 | 21% | **42** |
|  |  | Low-Mid | 22 | 88% |  | 0% | 3 | 12% | **25** |  | Yes | 14 | 54% | 2 | 8% | 10 | 38% | **26** |
|  |  | **All** | **41** | **63%** | **5** | **8%** | **19** | **29%** | **65** |  | **All** | **41** | **60%** | **8** | **12%** | **19** | **28%** | **68** |
| **Digital infra-structure** | **The lack of shared electronic health records** | High | 16 | 40% | 7 | 18% | 17 | 43% | **40** |  | No | 20 | 48% | 11 | 26% | 11 | 26% | **42** |
|  |  | Low-Mid | 17 | 74% | 1 | 4% | 5 | 22% | **23** |  | Yes | 13 | 50% | 2 | 8% | 11 | 42% | **26** |
|  |  | **All** | **33** | **52%** | **8** | **13%** | **22** | **35%** | **63** |  | **All** | **33** | **49%** | **13** | **19%** | **22** | **32%** | **68** |
| **Digital infra-structure** | **No system for the electronic transmission of a MOUD prescription from the prescriber to the pharmacy** | High | 17 | 44% | 3 | 8% | 19 | 49% | **39** |  | No | 24 | 57% | 7 | 17% | 11 | 26% | **42** |
|  |  | Low-Mid | 18 | 75% | 1 | 4% | 5 | 21% | **24** |  | Yes | 11 | 42% | 2 | 8% | 13 | 50% | **26** |
|  |  | **All** | **35** | **56%** | **4** | **6%** | **24** | **38%** | **63** |  | **All** | **35** | **51%** | **9** | **13%** | **24** | **35%** | **68** |
| **Digital infra-structure** | **The lack of an electronic drug monitoring system (to check for existing opioid prescribing and compliance with treatment)** | High | 13 | 33% | 5 | 13% | 21 | 54% | **39** |  | No | 26 | 62% | 9 | 21% | 7 | 17% | **42** |
|  |  | Low-Mid | 20 | 83% |  | 0% | 4 | 17% | **24** |  | Yes | 7 | 27% | 1 | 4% | 18 | 69% | **26** |
|  |  | **All** | **33** | **52%** | **5** | **8%** | **25** | **40%** | **63** |  | **All** | **33** | **49%** | **10** | **15%** | **25** | **37%** | **68** |
| **Health / clinical systems** | **There is limited benefit to the service or patients at this time** | High | 7 | 18% | 5 | 13% | 28 | 70% | **40** |  | No | 11 | 26% | 13 | 31% | 18 | 43% | **42** |
|  |  | Low-Mid | 8 | 32% | 6 | 24% | 11 | 44% | **25** |  | Yes | 4 | 15% | 1 | 4% | 21 | 81% | **26** |
|  |  | **All** | **15** | **23%** | **11** | **17%** | **39** | **60%** | **65** |  | **All** | **15** | **22%** | **14** | **21%** | **39** | **57%** | **68** |
| **Health / clinical systems** | **There are no incentives to providing TMOUD** | High | 15 | 39% | 9 | 24% | 14 | 37% | **38** |  | No | 25 | 60% | 12 | 29% | 5 | 12% | **42** |
|  |  | Low-Mid | 20 | 80% | 3 | 12% | 2 | 8% | **25** |  | Yes | 10 | 38% | 5 | 19% | 11 | 42% | **26** |
|  |  | **All** | **35** | **56%** | **12** | **19%** | **16** | **25%** | **63** |  | **All** | **35** | **51%** | **17** | **25%** | **16** | **24%** | **68** |
| **Health / clinical systems** | **There is no demand for it from patients** | High | 6 | 15% | 6 | 15% | 28 | 70% | **40** |  | No | 8 | 19% | 9 | 21% | 25 | 60% | **42** |
|  |  | Low-Mid | 6 | 24% | 2 | 8% | 17 | 68% | **25** |  | Yes | 4 | 15% | 2 | 8% | 20 | 77% | **26** |
|  |  | **All** | **12** | **18%** | **8** | **12%** | **45** | **69%** | **65** |  | **All** | **12** | **18%** | **11** | **16%** | **45** | **66%** | **68** |
| **Clinician perspectives** | **Clinicians believe it increases workload** | High | 4 | 10% | 11 | 28% | 25 | 63% | **40** |  | No | 7 | 17% | 15 | 36% | 20 | 48% | **42** |
|  |  | Low-Mid | 6 | 24% | 6 | 24% | 13 | 52% | **25** |  | Yes | 3 | 12% | 5 | 19% | 18 | 69% | **26** |
|  |  | **All** | **10** | **15%** | **17** | **26%** | **38** | **58%** | **65** |  | **All** | **10** | **15%** | **20** | **29%** | **38** | **56%** | **68** |
| **Clinician perspectives** | **Clinicians believe it is unsafe** | High | 12 | 30% | 13 | 33% | 15 | 38% | **40** |  | No | 18 | 43% | 16 | 38% | 8 | 19% | **42** |
|  |  | Low-Mid | 13 | 52% | 5 | 20% | 7 | 28% | **25** |  | Yes | 7 | 27% | 5 | 19% | 14 | 54% | **26** |
|  |  | **All** | **25** | **38%** | **18** | **28%** | **22** | **34%** | **65** |  | **All** | **25** | **37%** | **21** | **31%** | **22** | **32%** | **68** |
| **Clinician perspectives** | **Clinicians lack the necessary experience or training** | High | 17 | 43% | 9 | 23% | 14 | 35% | **40** |  | No | 25 | 60% | 10 | 24% | 7 | 17% | **42** |
|  |  | Low-Mid | 18 | 72% | 2 | 8% | 5 | 20% | **25** |  | Yes | 10 | 38% | 4 | 15% | 12 | 46% | **26** |
|  |  | **All** | **35** | **54%** | **11** | **17%** | **19** | **29%** | **65** |  | **All** | **35** | **51%** | **14** | **21%** | **19** | **28%** | **68** |
| **Clinician perspectives** | **There is no available training or way to gain the necessary experience for clinicians** | High | 17 | 43% | 13 | 33% | 10 | 25% | **40** |  | No | 21 | 50% | 11 | 26% | 10 | 24% | **42** |
|  |  | Low-Mid | 15 | 60% | 1 | 4% | 9 | 36% | **25** |  | Yes | 11 | 42% | 6 | 23% | 9 | 35% | **26** |
|  |  | **All** | **32** | **49%** | **14** | **22%** | **19** | **29%** | **65** |  | **All** | **32** | **47%** | **17** | **25%** | **19** | **28%** | **68** |
| **Consultations** | **Clinicians believe it is not possible to complete a full assessment without an in-person appointment** | High | 21 | 53% | 10 | 25% | 9 | 23% | **40** |  | No | 27 | 64% | 11 | 26% | 4 | 10% | **42** |
|  |  | Low-Mid | 19 | 76% | 2 | 8% | 4 | 16% | **25** |  | Yes | 13 | 50% | 4 | 15% | 9 | 35% | **26** |
|  |  | **All** | **40** | **62%** | **12** | **18%** | **13** | **20%** | **65** |  | **All** | **40** | **59%** | **15** | **22%** | **13** | **19%** | **68** |
| **Consultations** | **Concerns about communication difficulties for example hearing, vision impairment, language barriers** | High | 11 | 28% | 15 | 38% | 14 | 35% | **40** |  | No | 14 | 33% | 17 | 40% | 11 | 26% | **42** |
|  |  | Low-Mid | 11 | 44% | 4 | 16% | 10 | 40% | **25** |  | Yes | 8 | 31% | 5 | 19% | 13 | 50% | **26** |
|  |  | **All** | **22** | **34%** | **19** | **29%** | **24** | **37%** | **65** |  | **All** | **22** | **32%** | **22** | **32%** | **24** | **35%** | **68** |
| **Consultations** | **Concerns over privacy and confidentiality when consulting online** | High | 13 | 33% | 5 | 13% | 22 | 55% | **40** |  | No | 19 | 45% | 8 | 19% | 15 | 36% | **42** |
|  |  | Low-Mid | 12 | 48% | 3 | 12% | 10 | 40% | **25** |  | Yes | 6 | 23% | 3 | 12% | 17 | 65% | **26** |
|  |  | **All** | **25** | **38%** | **8** | **12%** | **32** | **49%** | **65** |  | **All** | **25** | **37%** | **11** | **16%** | **32** | **47%** | **68** |
| **Consultations** | **Reduced patient-provider rapport and trust without face-to-face contact** | High | 21 | 54% | 3 | 8% | 15 | 38% | **39** |  | No | 22 | 52% | 8 | 19% | 12 | 29% | **42** |
|  |  | Low-Mid | 12 | 48% | 4 | 16% | 9 | 36% | **25** |  | Yes | 11 | 42% | 3 | 12% | 12 | 46% | **26** |
|  |  | **All** | **33** | **52%** | **7** | **11%** | **24** | **38%** | **64** |  | **All** | **33** | **49%** | **11** | **16%** | **24** | **35%** | **68** |
| **Evidence base** | **There is insufficient evidence of its effectiveness and safety** | High | 11 | 28% | 14 | 35% | 15 | 38% | **40** |  | No | 18 | 43% | 15 | 36% | 9 | 21% | **42** |
|  |  | Low-Mid | 14 | 56% | 2 | 8% | 9 | 36% | **25** |  | Yes | 7 | 27% | 4 | 15% | 15 | 58% | **26** |
|  |  | **All** | **25** | **38%** | **16** | **25%** | **24** | **37%** | **65** |  | **All** | **25** | **37%** | **19** | **28%** | **24** | **35%** | **68** |

# Survey instrument


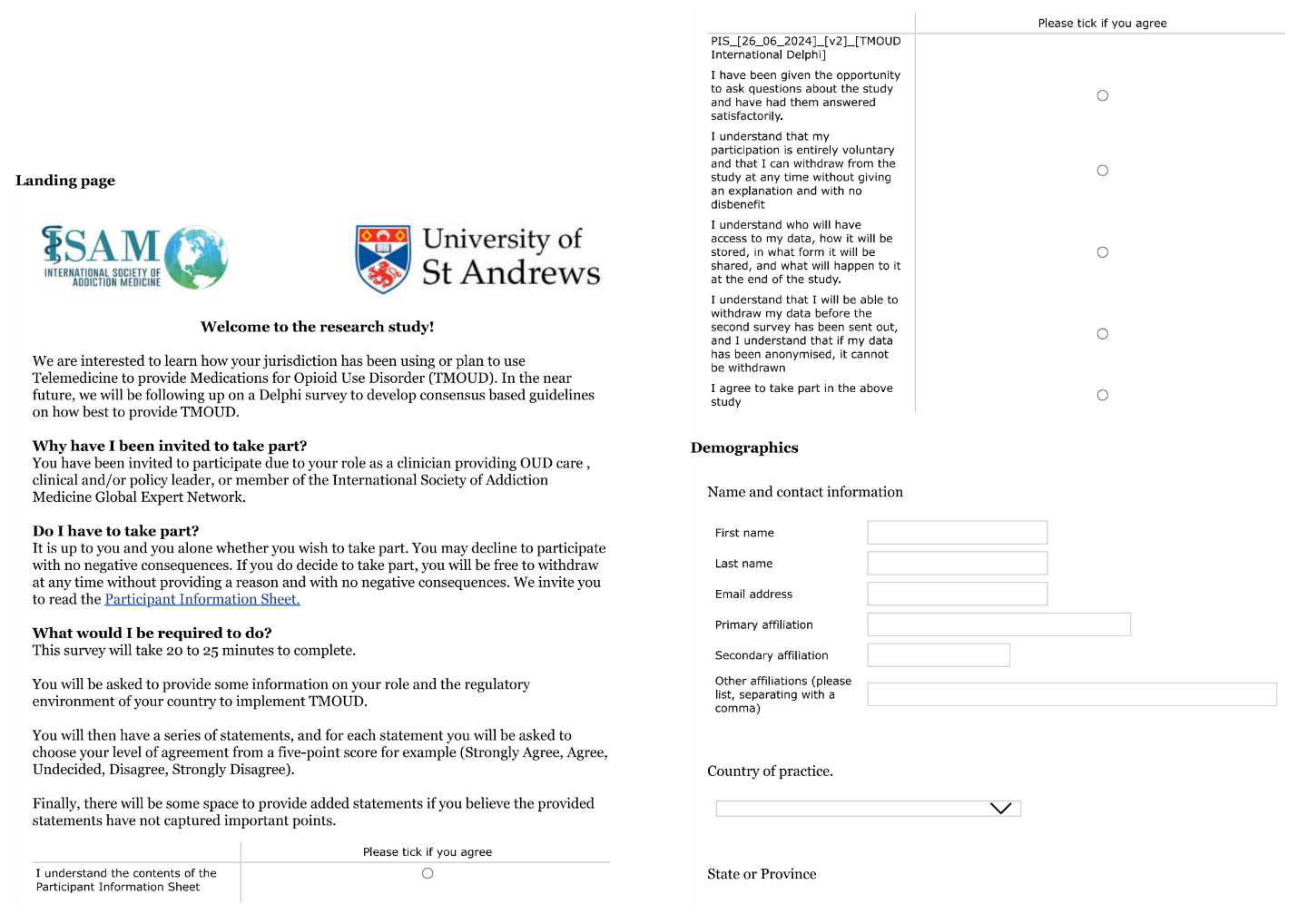


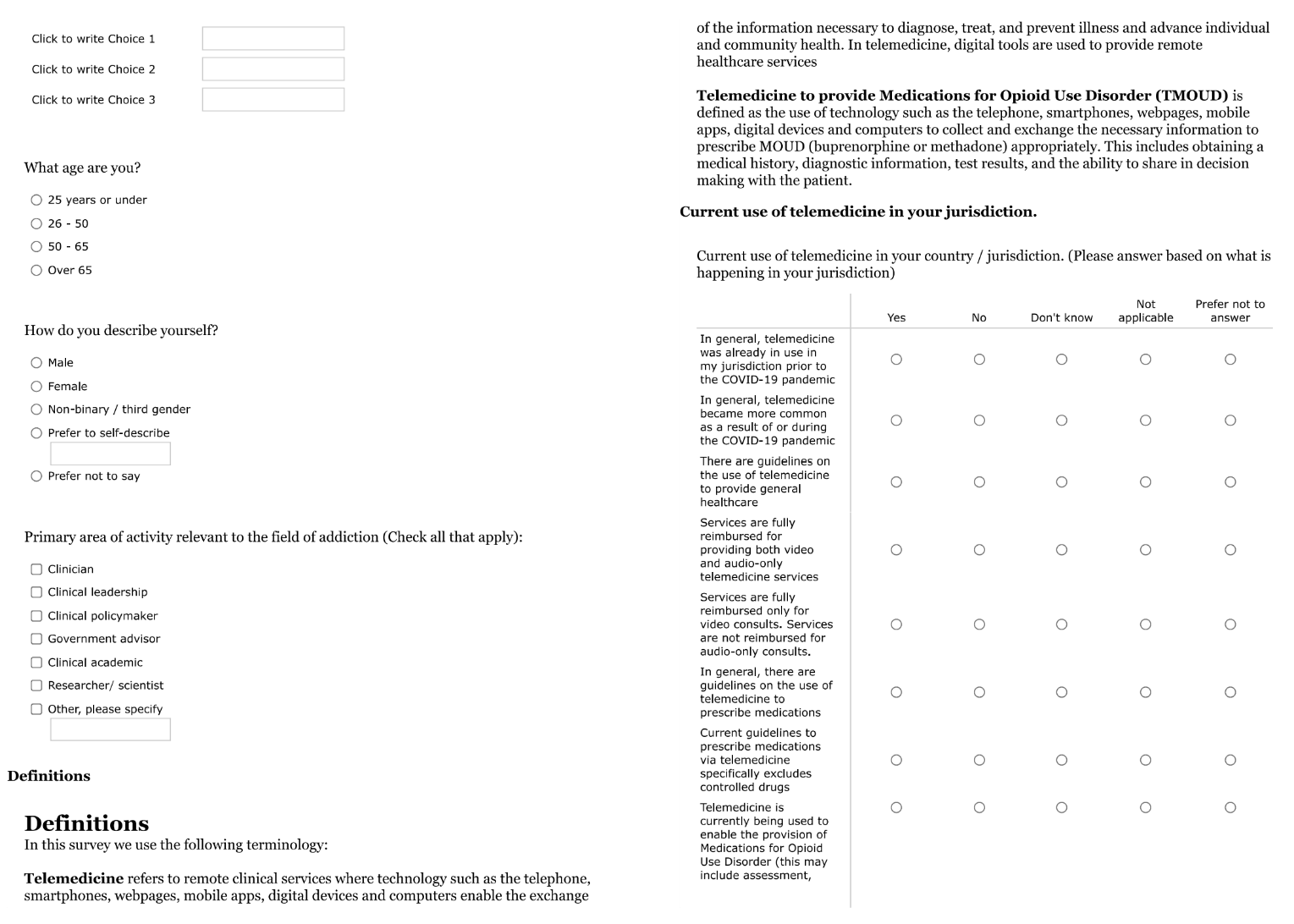


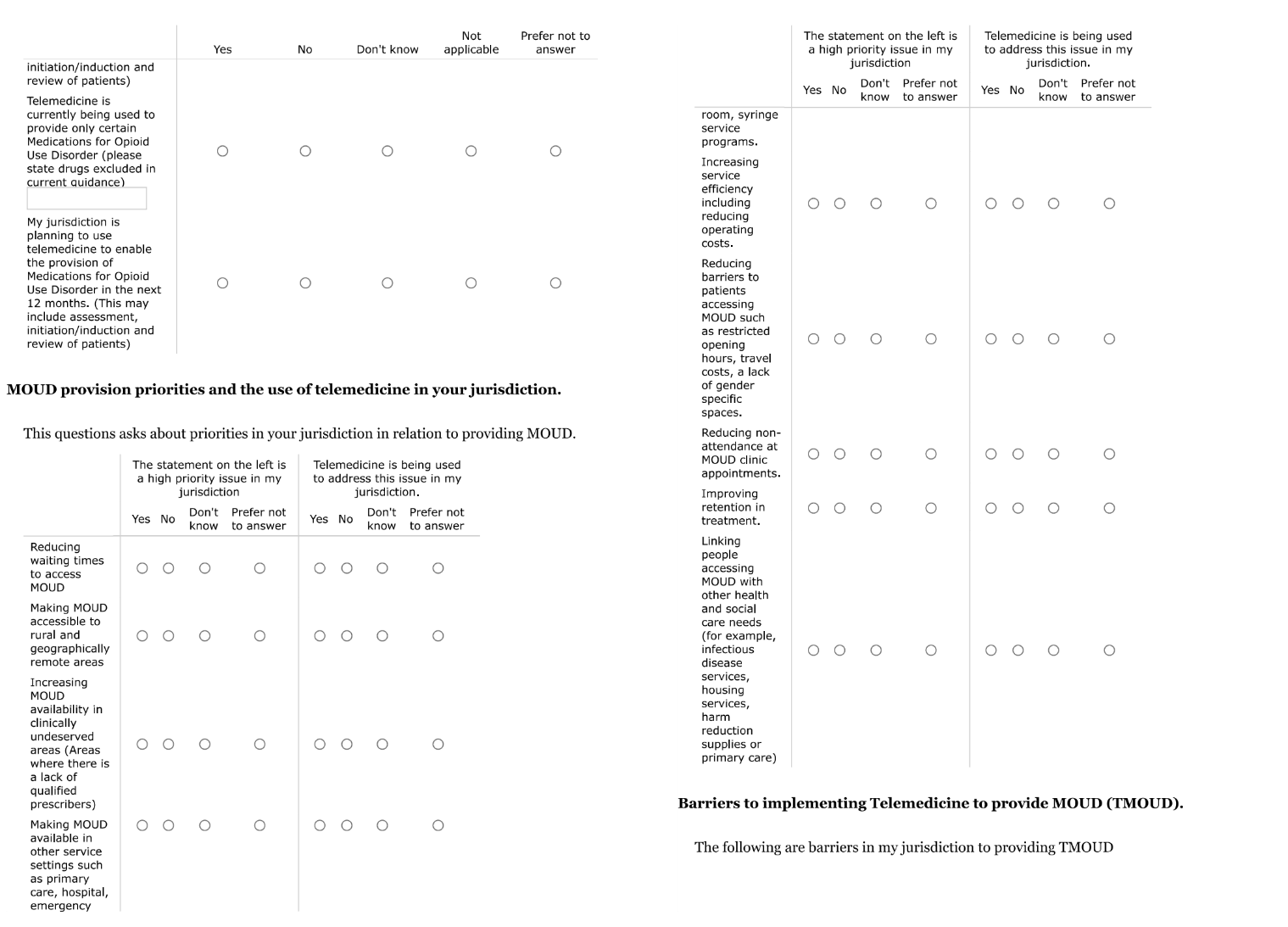


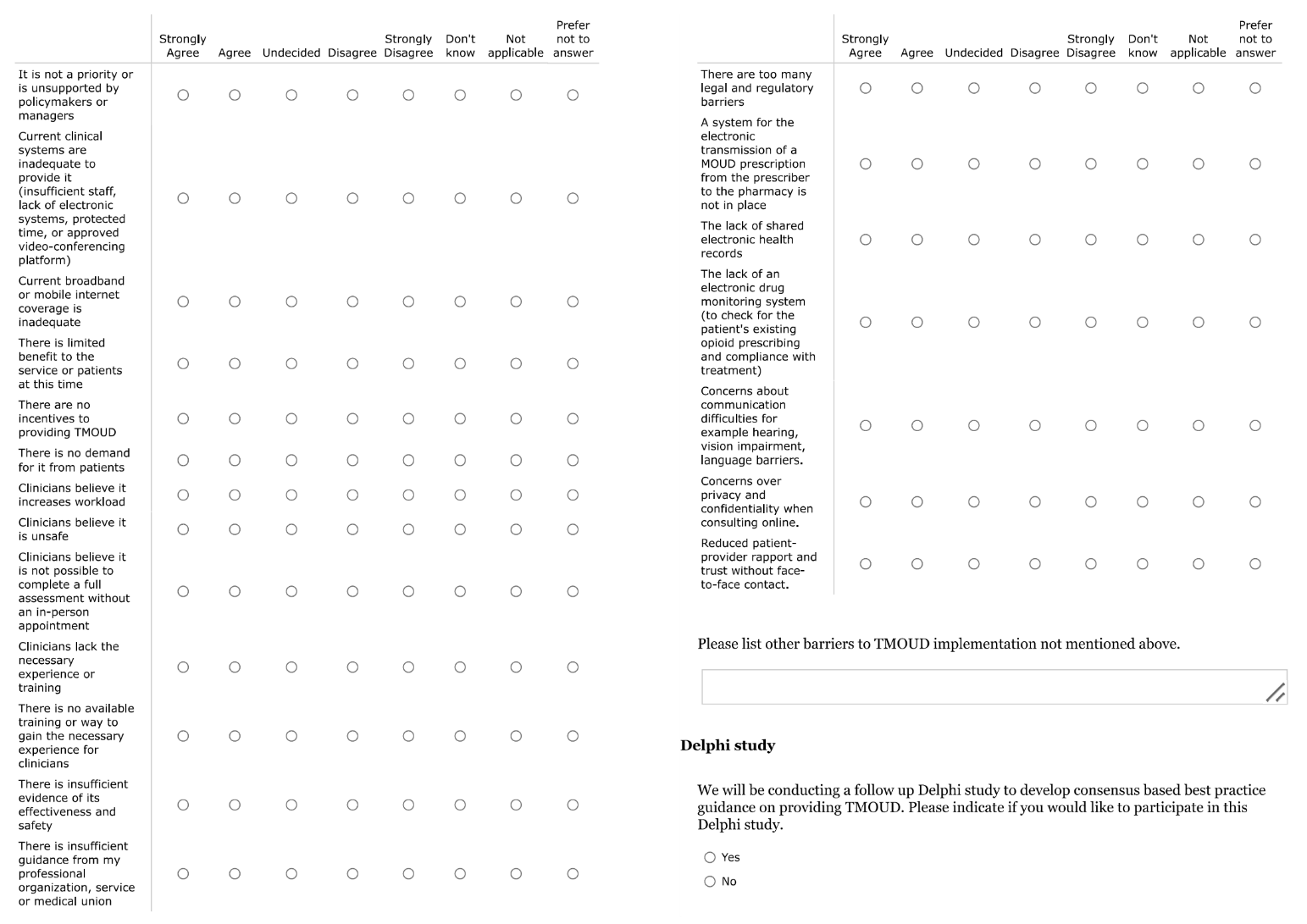

Supplement: sj-docx-1-jtt-10.1177_1357633X251394442 - Supplemental material for Global perspectives on telemedicine-enabled medications for opioid use disorder: Practices, priorities, and barriers [file sj-docx-1-jtt-10.1177_1357633X251394442.docx]
